# Supplementary material for: A Bifunctional Electrocatalyst for OER and ORR based on a Cobalt(II) Triazole Pyridine Bis‐[Cobalt(III) Corrole] Complex
Source: Angew Chem Weinheim Bergstr Ger. 2023 Apr 13;135(21):e202302208. doi: 10.1002/ange.202302208 (PMC10952570; doi:10.1002/ange.202302208)
Supplement: Supplementary file 1 — Supporting Information [file ANGE-135-0-s001.pdf]

Angewandte Chemie
Eine Zeitschrift der Gesellschaft Deutscher Chemiker

Supporting Information

A Bifunctional Electrocatalyst for OER and ORR based on a Cobalt(II) Triazole Pyridine Bis-[Cobalt(III) Corrole] Complex

*A. Aljabour, H. Awada, L. Song, H. Sun, S. Offenthaler, F. Yari, M. Bechmann, M. C. Scharber, W. Schöfberger**

Table of Contents

1. Materials and methods **3**
2. Synthesis procedures **4**
3. Analytical Data 8
4. Electrochemical Characterization **19**
5. References 26

1. Materials and methods

Chemicals were purchased from Fluka, Alfa Aesar, Sigma-Aldrich, and Merck. Pyrrole was freshly distilled before use. 5-(4-tert-Butylphenyl)-dipyrromethane [1], 3-triisopropylsilyl-propynal [2], and 2-(azidomethyl)pyridine [3]were synthesized according to reported literature procedures. DCM was obtained using a M. Braun Inert Gas System GmBH where it is stored over molecular sieve MB-SPS-7 under argon atmosphere. NMR solvents were purchased from Sigma-Aldrich. TLC was performed on Macherey-Nagel silica gel 60 (0.20 mm) with fluorescent indicator UV254 on aluminium plates and on Merck aluminium oxide 60 (0.20 mm) with fluorescent indicator UV254 on aluminium plates. For chromatography, silica-gel columns were prepared with silica-gel 60 (0.070-0.20 mesh) from Grace and aluminium oxide 60, basic, activity level II from Acros. ^{1}H and ^{13}C NMR spectra were recorded on a Bruker DRX 500 MHz spectrometer and a Bruker Advance 300 MHz NMR spectrometer. Chemical shifts are given in parts per million (ppm) on the delta scale (δ) and are referenced to the used deuterated solvent for ^{1}H-NMR. High resolution mass spectra were obtained using an Agilent 6520 Q-TOF mass spectrometer with an ESI source and an Agilent G1607A coaxial sprayer and a Thermo Fisher Scientific LTQ Orbitrap XL with an Ion Max API Source. MALDI-TOF was measured on a . UV-Vis absorption spectra were collected on a Varian CARY 300 Bio spectrophotometer from 200 to 900 nm.

2. Synthesis procedures

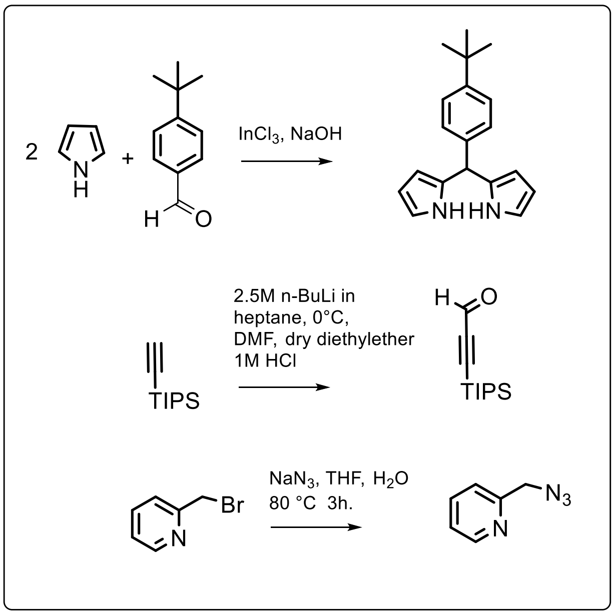

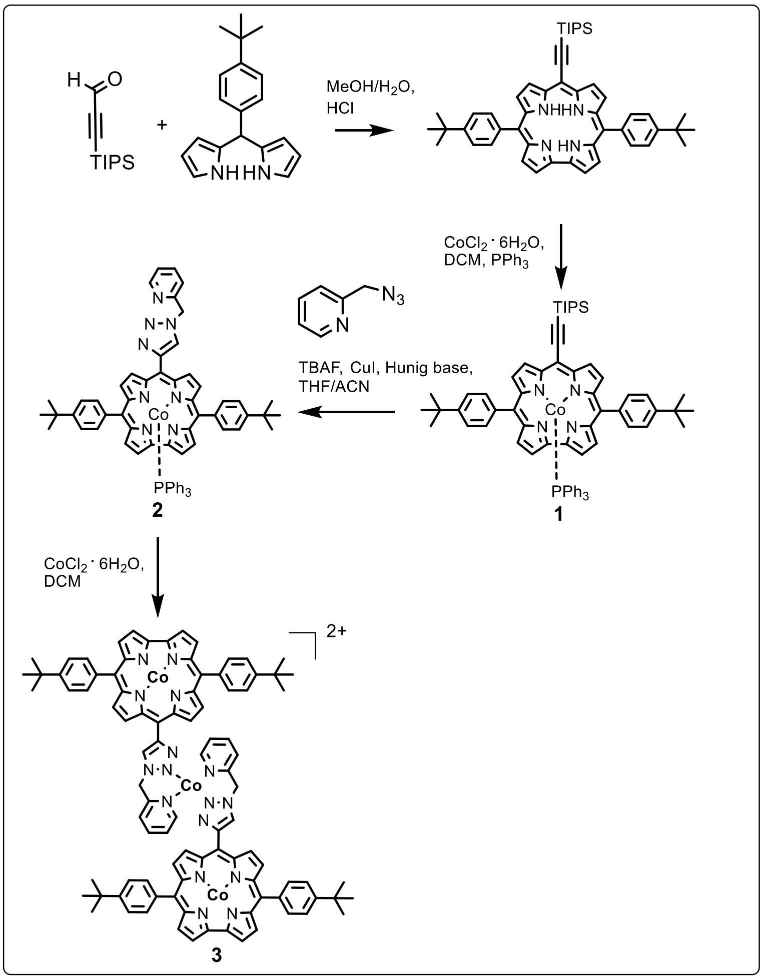

Synthesis of 5-(4-*t*-Butylphenyl)-dipyrromethane:

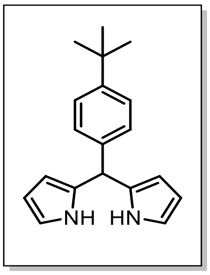

4-*t*-butylbenzaldehyde (0.9234 g, 5.7 mmol), and pyrrole (39.5 ml, 570 mmol) were mixed under inert atmosphere. $InCl_3$ (0.154 g, 5.7mmol) was added and the mixture was stirred for 2h at room temperature. After that, powdered NaOH (0.7 g) was added and the mixture was stirred for an additional 1h, the mixture was diluted with heptane and evaporated to dryness to obtain the product as off white powder (1.39 g, 87%), ^{1}H-NMR (300 MHz, $CDCl_3$, 25 °C): δ=7.92(s, br, 2H, N-H), 7.328(d, 2H, phenyl), 7.143(d, 2H, phenyl), 6.69(dd, 2H, pyrrole), 6.155(dd, 2H, pyrrole), 5.94(t, 2H, pyrrole), 5.45(s, 1H, CH), 1.3(s, 9H, t-butyl).

Synthesis of 3-triisopropylsilyl-propynal:

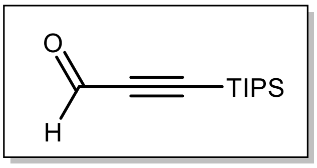

(Triisopropylsilyl)-acetylene (1.16ml, 5mmol) was dissolved in 5ml dry ether under inert atmosphere at 0°C, then 2.5M n-BuLi solution in heptane (2ml, 5mmol) was added dropwise. After 30 minutes, DMF (1.55ml, 20mmol) dissolved in 5 ml dry ether was added dropwise also at 0 °C and the reaction was stirred for an additional 1h after which the reaction was quenched with 8 ml 1M HCl. The mixture was extracted with more dry ether, then the organic fraction was dried under Na_2SO_4, and solvents removed under reduced pressure. The dried product is a yellowish oil that is purified via flash column chromatography (silica, Heptane/EtOAC, [20:1]) to afford the product as clear oil (0.94g, 90%). R_f=0.56, ^{1}H-NMR (300 MHz, $CDCl_3$, 25 °C): δ= 9.14(s, 1H), 1.05(s, 21H).

Synthesis of 2-(azidomethyl)-pyridine:

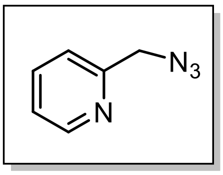

2-(bromomethyl)-pyridine hydrobromide (0.25 g, 1 mmol) was dissolved in 4 ml THF and mixed with a solution of NaN_3 (0.13 g, 2 mmol) and H_2O. The mixture is refluxed at 80 °C for 3h. when the reaction is finished, the mixture is diluted with EtOAC, washed with brine and water, organic phase dried with Na_2SO_4, filtered and solvents evaporated under reduced pressure to afford the product as dark brown oil (75 mg. 50%), ^{1}H-NMR (300 MHz, $CDCl_3$, 25°C): δ= 8.45(d, 1H, pyridine), 7.57(td, 1H, pyridine), 7.19(d, 1H, pyridine), 7.1(m, 1H, pyridine), 4.34(s, 2H, CH_2).

Synthesis of 5,15-4-*t*-butylphenyl-10-triisopropylsilylethynyl-corrole :

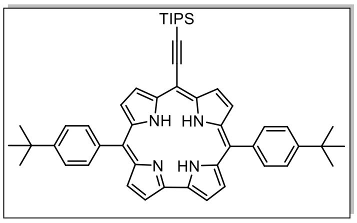

1 (500 mg, 1.80 mmol) and **2** (190mg, 0.90 mmol) were dissolved in a mixture of MeOH (100 mL), H_2O (94 mL), and HCl (6 mL, 36 %). The mixture was stirred for 2h at room temperature, then it was extracted with DCM and dried over Na_2SO_4. The mixture is diluted until the overall volume of DCM was 250 ml and *p*-chloranil (0.33g, 1.34mmol) is added. The solution is stirred overnight, evaporated to dryness, and purified via column chromatography (silica, DCM/Heptane, [1:2]) to obtain the product as green solid (100mg, 15%). R_f=0.6, ^{1}H-NMR (300 MHz, $CDCl_3$, 25°C): δ= 9.26(d, 2H, pyrrole), 8.92(d, 2H, pyrrole), 8.82(d,2H, pyrrole), 8.51(s, 2H, pyrrole), 8.26(d, 4H, phenyl), 7.82(d, 4H, phenyl), 7.4-7.6(m, 3H, pyrrole N-H), 1.58(s, 18H, t-butyl), 1.43(s, 21H, TIPS). *m/z*: calcd. for C50H_{59}N4Si^+: 743.4509; found $[M+H]^+$: 743.5. λ_{max} nm: 281, 430, 584, 628.[4]

Synthesis of cobalt (III) 5,15-4-*t*-butylphenyl-10-triisopropylsilylethynyl-corrole (1):

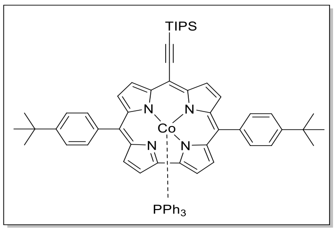

5,15-4-*t*-butylphenyl-10-triisopropylsilylethynyl-corrole (120 mg, 0.16 mmol) was dissolved in 20 ml DCM under inert atmosphere. $Co(OAc)_2.4H_2O$ (115 mg, 0.65 mmol) dissolved in MeOH and PPh_3 (0.16 mmol, 42 mg) was added to the reaction mixture and the solution was refluxed until consumption of starting materials. The mixture is diluted with DCM and washed with H_2O thrice. Organic fraction was collected, dried with Na_2SO_4 and solvents evaporated under reduced pressure. Purification with column chromatography (silica, DCM/Heptane, [1:2]) to obtain the product as red solid (150 mg, 87%) R_f=0.55, ^{1}H-NMR (300 MHz, $CDCl_3$, 25°C): δ= 8.78(d, 2H, pyrrole), 8.72(d, 2H, pyrrole), 8.43(d,2H, pyrrole), 8.18(s, 2H, pyrrole), 7.65(m, 4H, phenyl), 7.5(m, 4H, phenyl), 7.07(t, 3H, PPh_3), 6.7(t, 6H, PPh_3), 4.77(m, 6H, PPh_3) 1.56(s, 18H, t-butyl), 1.4(s, 21H, TIPS). *m/z*: calcd. for $C_{68}H_{70}CoN_4Psi^+$: 1060.4439; found $[M+H]^+$: 1061.45. λ_{max} nm: 402, 541.

Synthesis of cobalt (III) 5,15-4-*t*-butylphenyl-10-{4-[-(2-methylpyridine)-1,2,3-triazole]}-corrole (2):

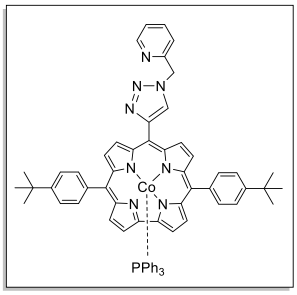

1 (200 mg, 0.19 mmol), 1M TBAF in heptane (0.3 ml, 0.3 mmol), CuI (38 mg, 2mmol), **3** (30 mg, 22 mmol), and Hunig base (33 µl, 0.19 mmol) were mixed in MeCN/THF (1:1). And the mixture was stirred at 40°C. the mixture was monitored via TLC until the consumption of starting material. The mixture is diluted with DCM and washed with H_2O thrice. Organic fraction was collected, dried with Na_2SO_4 and solvents evaporated under reduced pressure Purification with column chromatography (Alumina, solvent polarity was increased starting from DCM/Heptane (1:1) and then DCM/EtOAC (9:1) to obtain the product as a red powder (140 mg, 71.5%). ^{1}H-NMR (500 MHz, $CDCl_3$, 25°C): δ= 8.71(d, 1H, pyridine), 8.62(d, 2H), 8.38(d, 2H), 8.33(d,2H,), 8.12(d, 2H, pyrrole), 8.02(m, 3H), 7.82(td, 1H, pyridine), 7.64-7.53(m.6H), 7.46(d, 1H, pyridine), 7.35(td, 1H, pyridine), 7.(t, 3H, PPh_3), 6.67(t, 6H, PPh_3), 5.96(s,2H, CH_2), 4.69(m, 6H, PPh_3), 1.54(s, 18H, t-butyl). ^{13}C-NMR (125 MHz, $CDCl_3$, 25°C): δ_C, ppm: 155, 150, 146, 145, 144, 138, 137, 136, 131, 130, 129, 127, 124, 123, 122, 118, 55, 35, 31, 29. HRMS *m*/*z*: calcd. for $C_{65}H_{57}CoN_8P^+$: 1039.3776; found $[M+H]^+$: 1039.3774. λ_{max} nm: 393, 560.

Synthesis of Co (II) [cobalt (III) 5,15-4-*t*-butylphenyl-10-{4-[-(2-methylpyridine)-1,2,3-triazole]}-corrole]$_2$ (3):

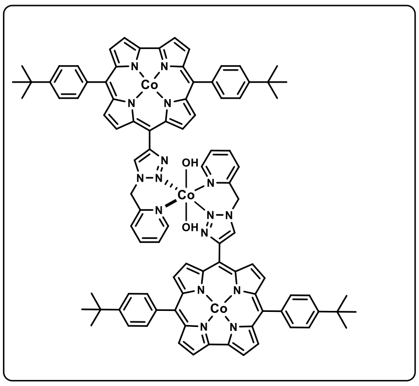

50 mg of **2** (0.047 mmol) were dissolved in minimum amount of MeOH, then $CoCl_2$ (6 mg, 0.024 mmol) was added, and the mixture was stirred for 6 days in the dark. The mixture is diluted with DCM and washed with H_2O thrice. Organic fraction was collected, dried with Na_2SO_4 and solvents evaporated under reduced pressure Purification with column chromatography (Alumina, solvent polarity was increased starting from DCM/Heptane (1:1) and then DCM/EtOAC (9:1) to DCM/MeOH (9:1) to obtain the product as an orange powder (23 mg, 0.014 mmol). Due to the presence of Co (II), NMR spectra possess paramagnetic shift and enhanced relaxation. MALDI-TOF *m*/*z*: calcd. For $C_{94}H_{83}Co_3N_{16}^+$: 1612.4983; found $[M+H]^+$: 1612.519, $[M]^{2+}$: 806.212 λ_{max} nm: 386, 444, 517, 551, 767, 840.[5]

3. Analytical Data

5-(4-tert-Butylphenyl)-dipyrromethane (1):

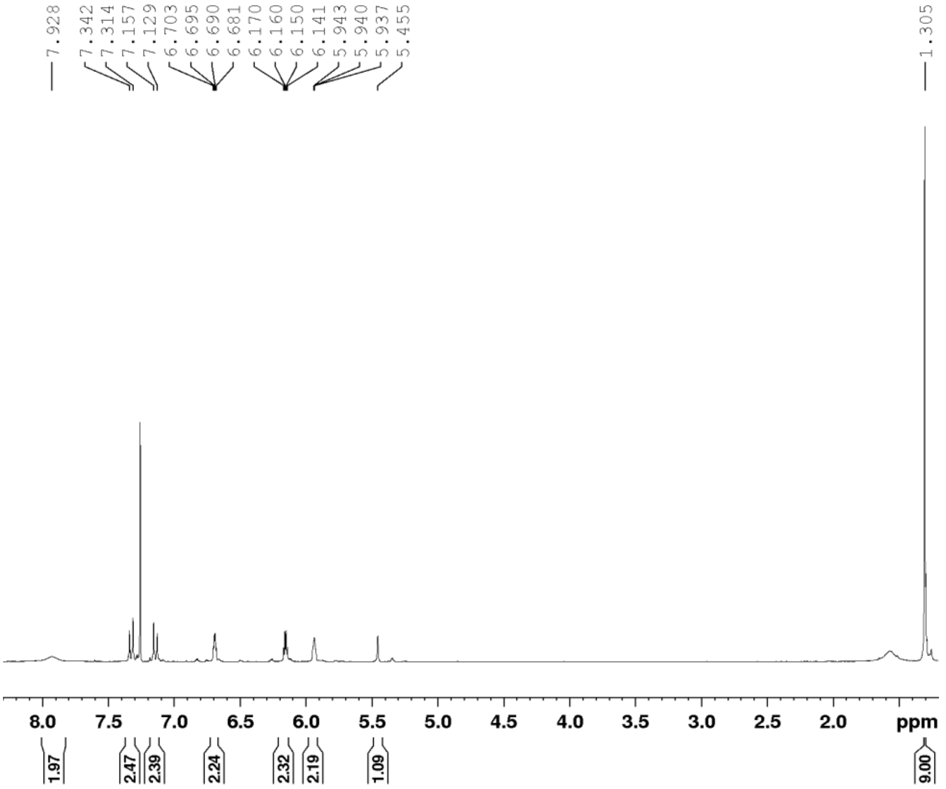

Figure S1. ^{1}H-NMR spectrum of 5-(4-*t*-Butylphenyl)-dipyrromethane in $CDCl_3$.

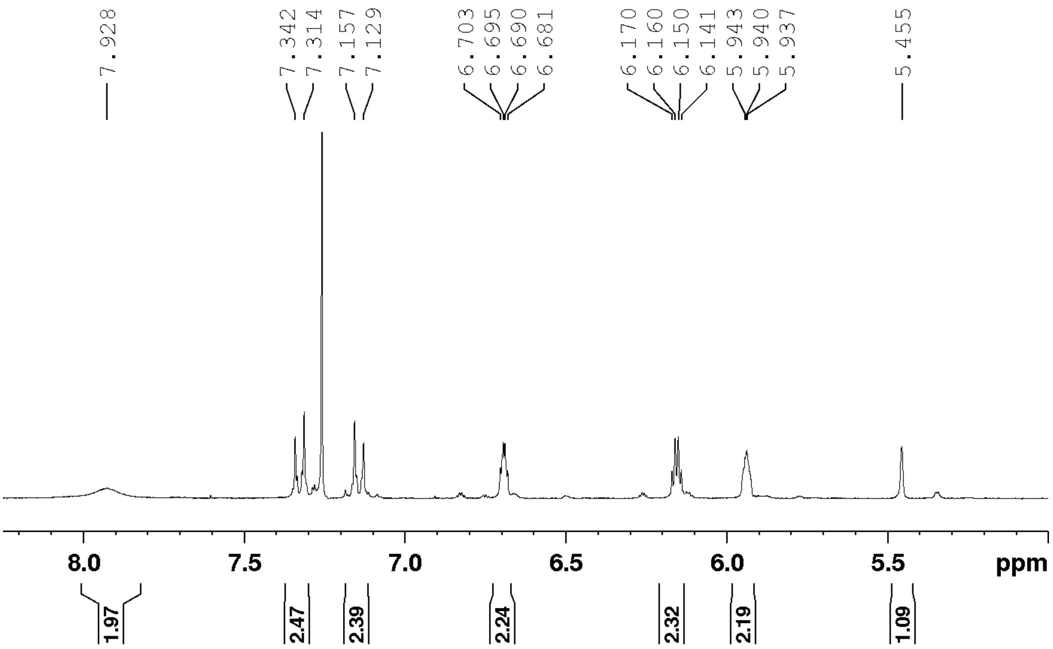

Figure S2. Zoomed ^{1}H-NMR spectrum of 5-(4-*t*-Butylphenyl)-dipyrromethane in $CDCl_3$.

2-triisopropylsilyl-propynal (2):

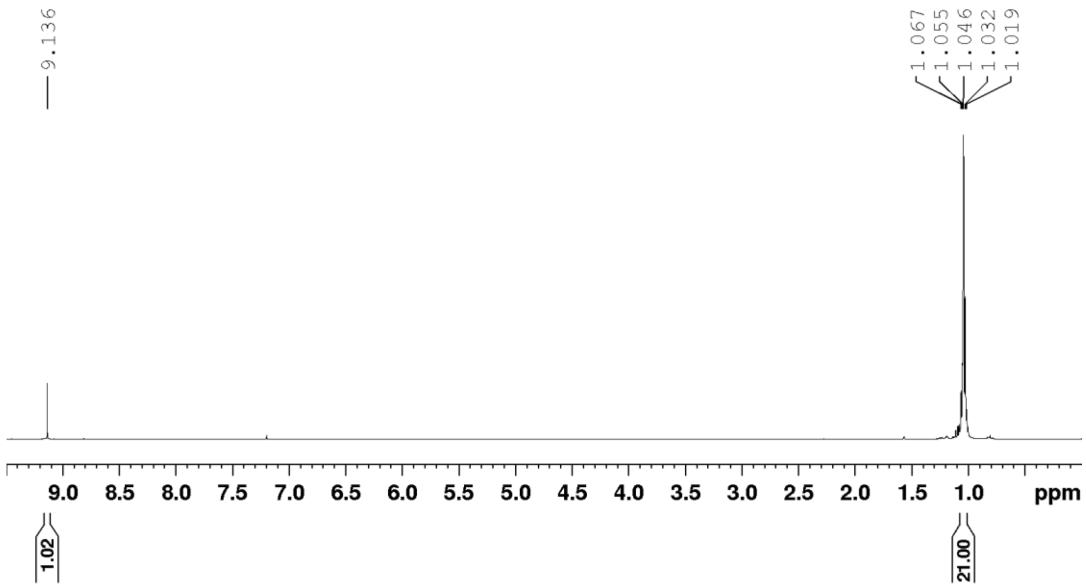

Figure S3. ^{1}H-NMR spectrum of 3-triisopropylsilyl-propynal in $CDCl_3$.

3-(azidomethyl)-pyridine (3):

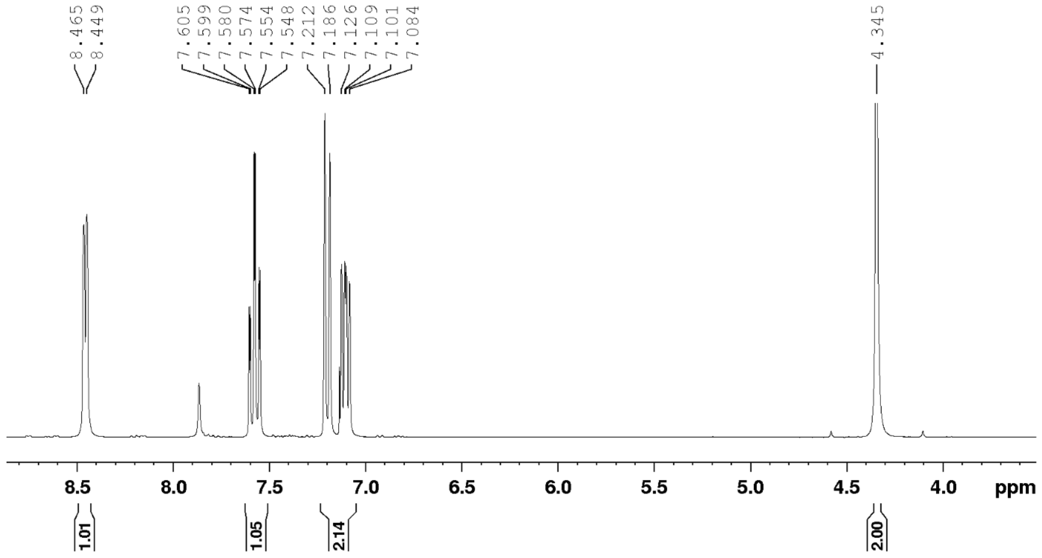

Figure S4. ^{1}H-NMR spectrum of 2-(azidomethyl)-pyridine in $CDCl_3$.

5,15-4-*t*-butylphenyl-10-triisopropylsilylethynyl-corrole (4):

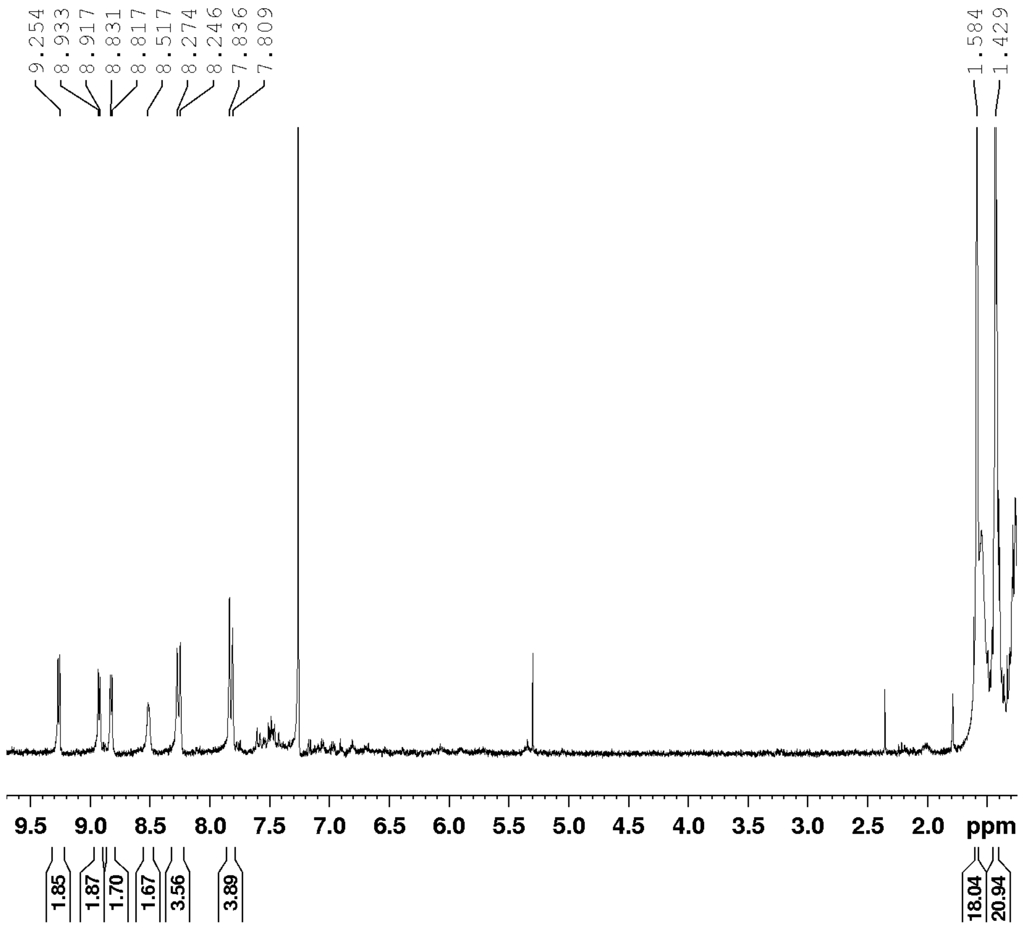

Figure S5. ^{1}H-NMR spectrum of 5,15-4-*t*-butylphenyl-10-triisopropylsilylethynyl-corrole in $CDCl_3$.

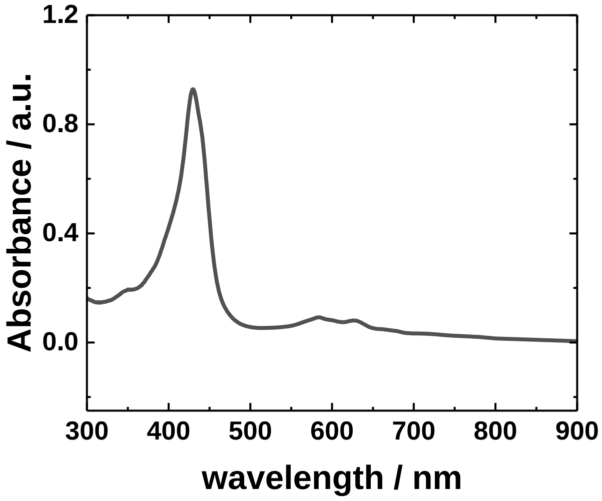

Figure S6. UV-VIS spectrum of 5,15-4-*t*-butylphenyl-10-triisopropylsilylethynyl-corrole in CH_2Cl_2.

Cobalt (III) 5,15-4-*t*-butylphenyl-10-triisopropylsilylethynyl-corrole (5):

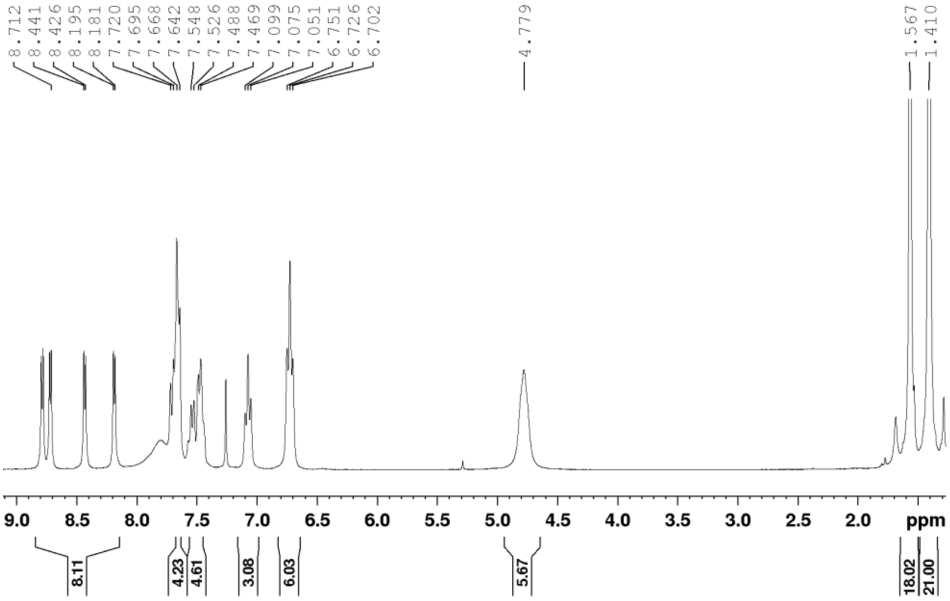

Figure S7. ^{1}H-NMR spectrum of cobalt (III) 5,15-4-*t*-butylphenyl-10-triisopropylsilylethynyl-corrole in $CDCl_3$.

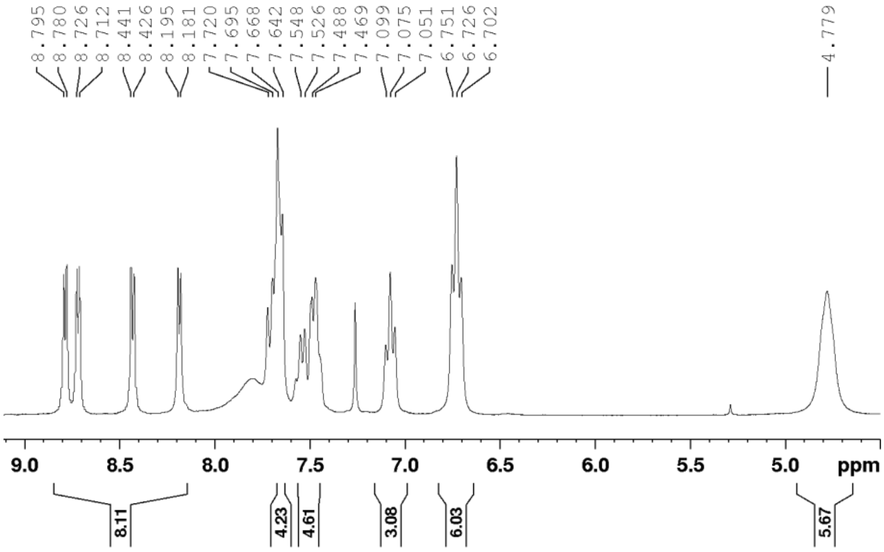

Figure S8. Zoomed ^{1}H-NMR spectrum of cobalt(III) 5,15-4-*t*-butylphenyl-10-triisopropyl-silylethynyl-corrole in $CDCl_3$.

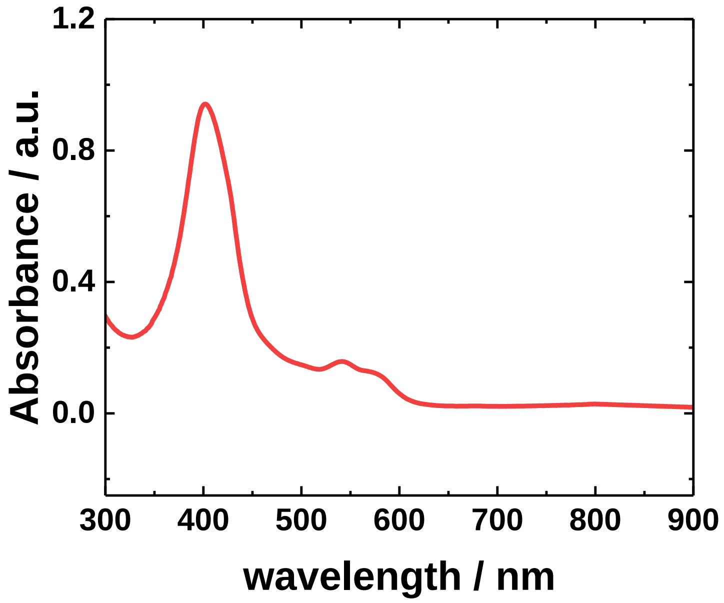

Figure S9. UV-VIS spectrum of cobalt (III) 5,15-4-*t*-butylphenyl-10-triisopropylsilylethynyl-corrole in CH_2Cl_2.

Cobalt (III) 5,15-4-*t*-butylphenyl-10-{4-[-(2-methylpyridine)-1,2,3triazole]}-corrole (6):

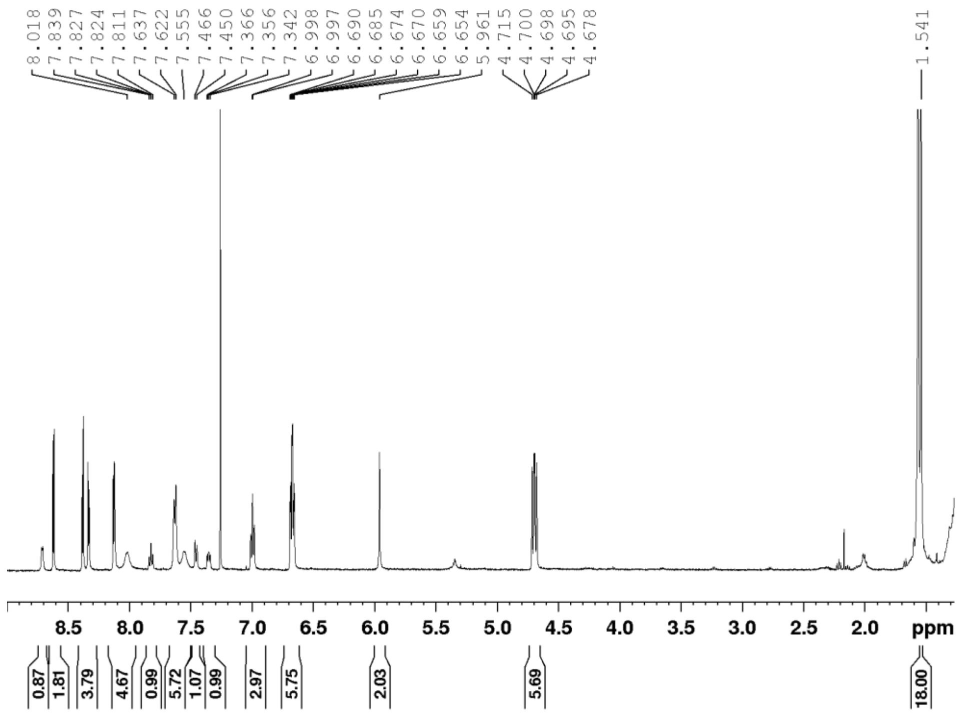

Figure S10. ^{1}H-NMR spectrum of cobalt (III) 5,15-4-*t*-butylphenyl-10-{4-[-(2-methylpyridine)-1,2,3-triazole]}-corrole in $CDCl_3$.

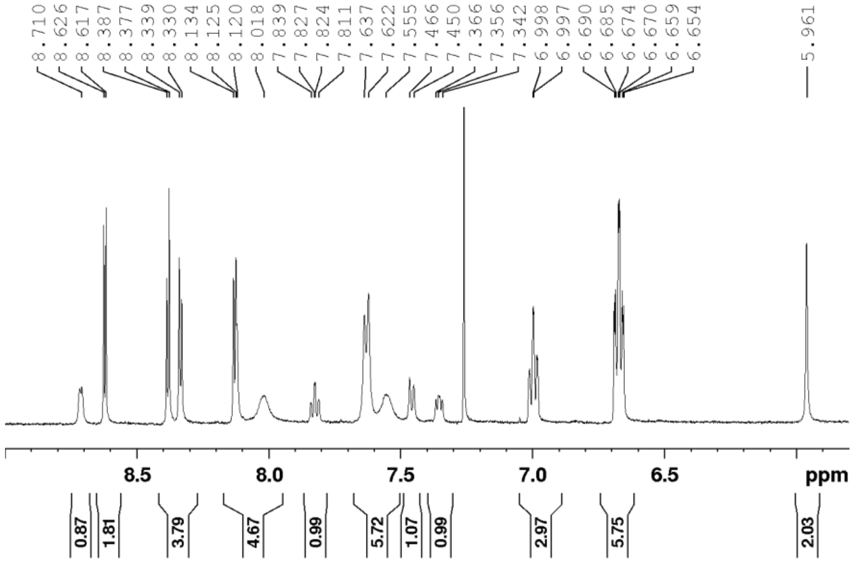

Figure S11. Zoomed ^{1}H-NMR spectrum of cobalt (III) 5,15-4-*t*-butylphenyl-10-{4-[-(2-methylpyridine)-1,2,3-triazole]}-corrole in $CDCl_3$.

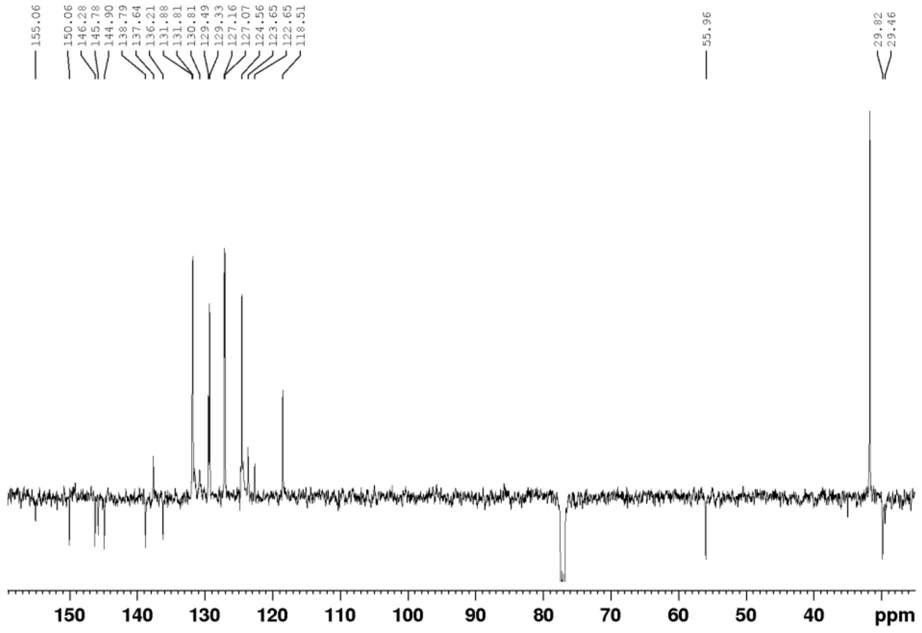

Figure S12. ^{13}C-NMR spectrum of cobalt (III) 5,15-4-*t*-butylphenyl-10-{4-[-(2-methylpyridine)-1,2,3-triazole]}-corrole in $CDCl_3$.

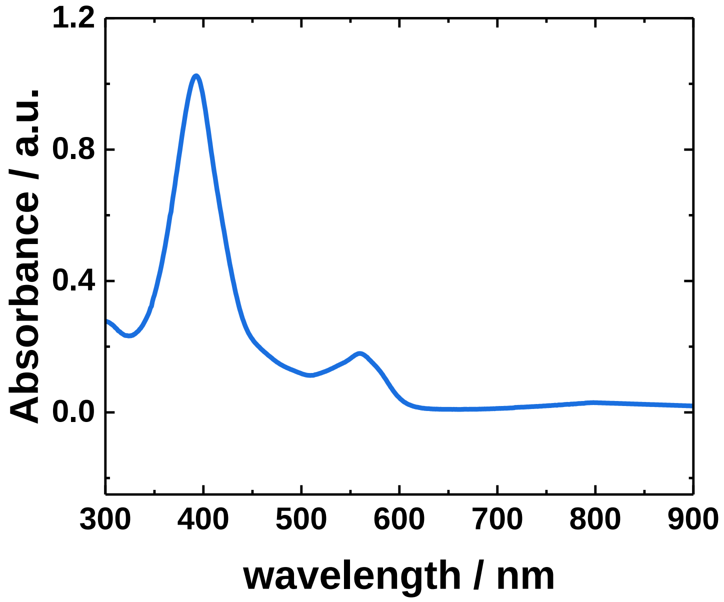

Figure S13. UV-VIS spectrum of cobalt (III) 5,15-4-*t*-butylphenyl-10-{4-[-(2-methylpyridine)-1,2,3-triazole]}-corrole in CH_2Cl_2.

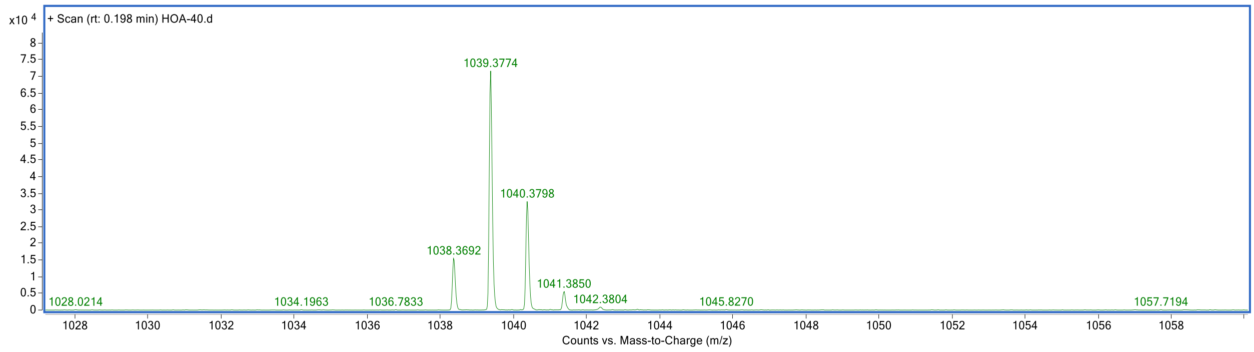

Figure S14. HRMS of cobalt (III) 5,15-4-*t*-butylphenyl-10-{4-[-(2-methylpyridine)-1,2,3-triazole]}-corrole.

Cobalt (II) [cobalt (III) 5,15-4-*t*-butylphenyl-10-{4-[-(2-methylpyridine)-1,2,3triazole]}-corrole]$_2$ (3):

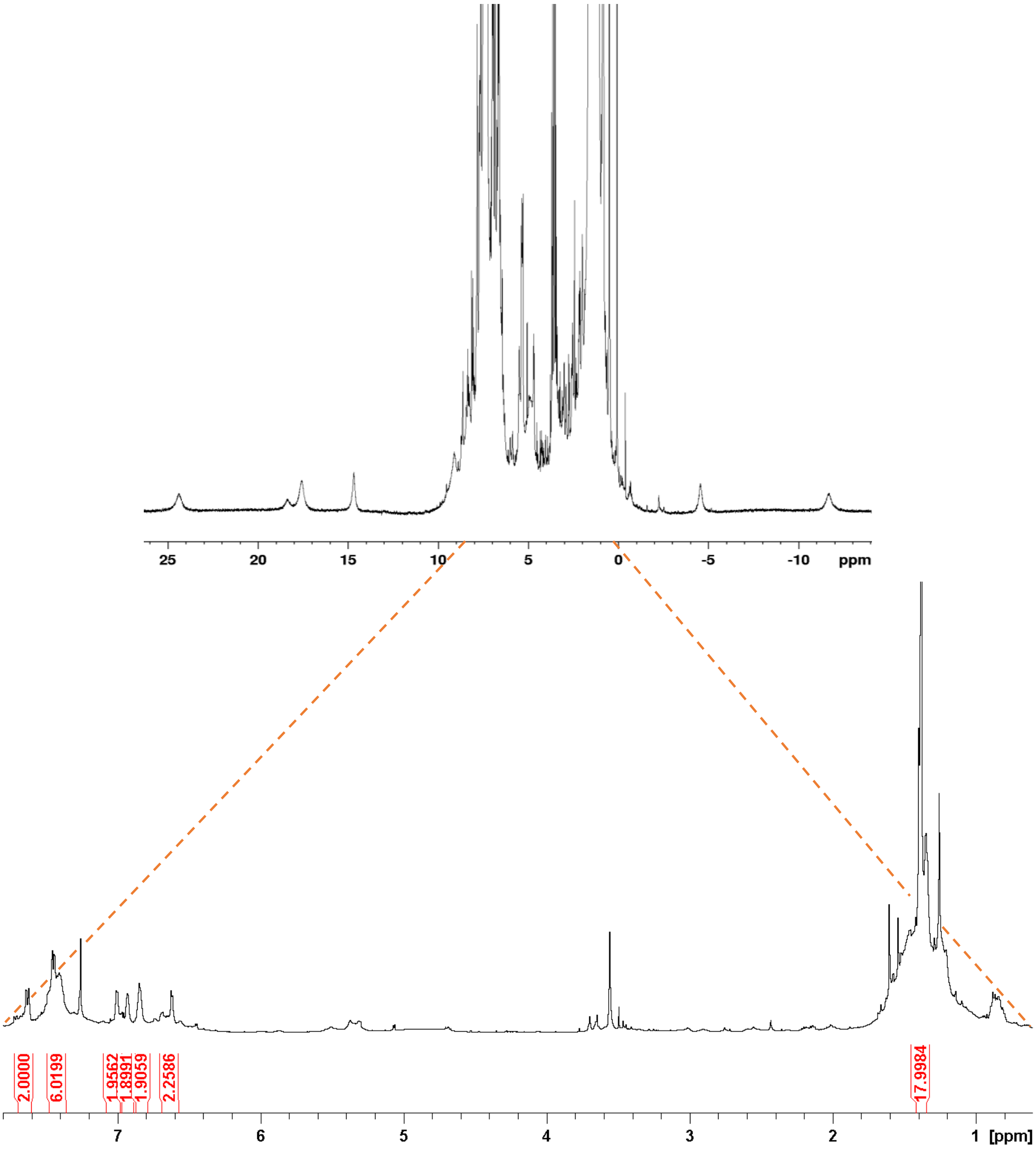

Figure S15. Top: Survey ^{1}H-NMR spectrum of cobalt (II) [cobalt (III) 5,15-4-*t*-butylphenyl-10-{4-[-(2-methylpyridine)-1,2,3-triazole]}-corrole]$_2$ in $CDCl_3$ (including paramagnetically shifted ^{1}H resonances of triazole pyridine subunit). Bottom: Zoomed ^{1}H NMR region of cobalt(III) corrole subunits (assignment of phenyl-H's, β-pyrrole H's and t-butyl-H's is possible, these protons are not affected by the pseudocontact shift).

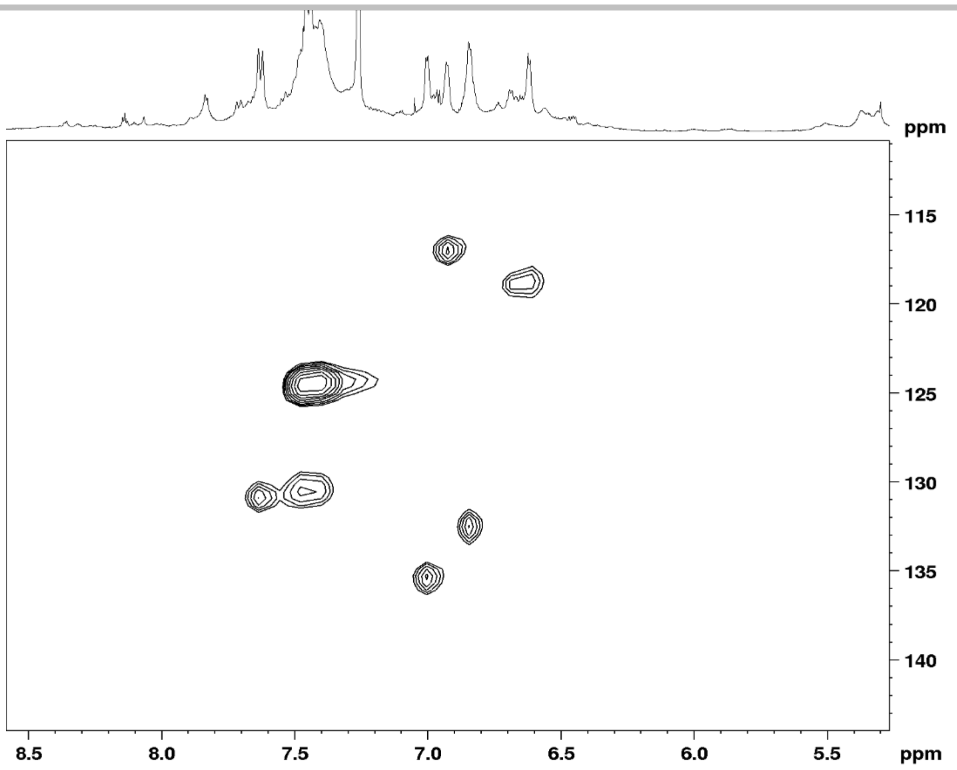

Figure S16. HSQC spectrum of cobalt (II) [cobalt (III) 5,15-4-*t*-butylphenyl-10-{4-[-(2-methylpyridine)-1,2,3-triazole]}-corrole]$_2$ in $CDCl_3$.

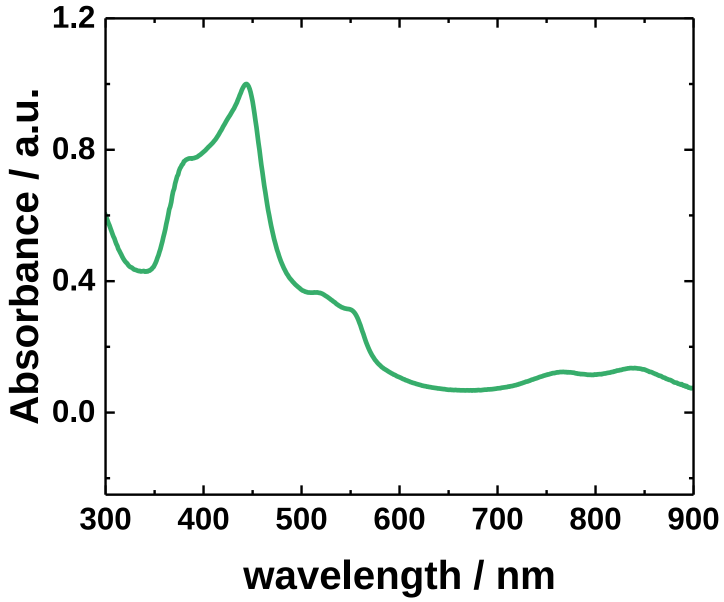

Figure S17. UV-vis spectrum of cobalt (II) [cobalt (III) 5,15-4-*t*-butylphenyl-10-{4-[-(2-methylpyridine)-1,2,3-triazole]}-corrole]$_2$ in CH_2Cl_2.

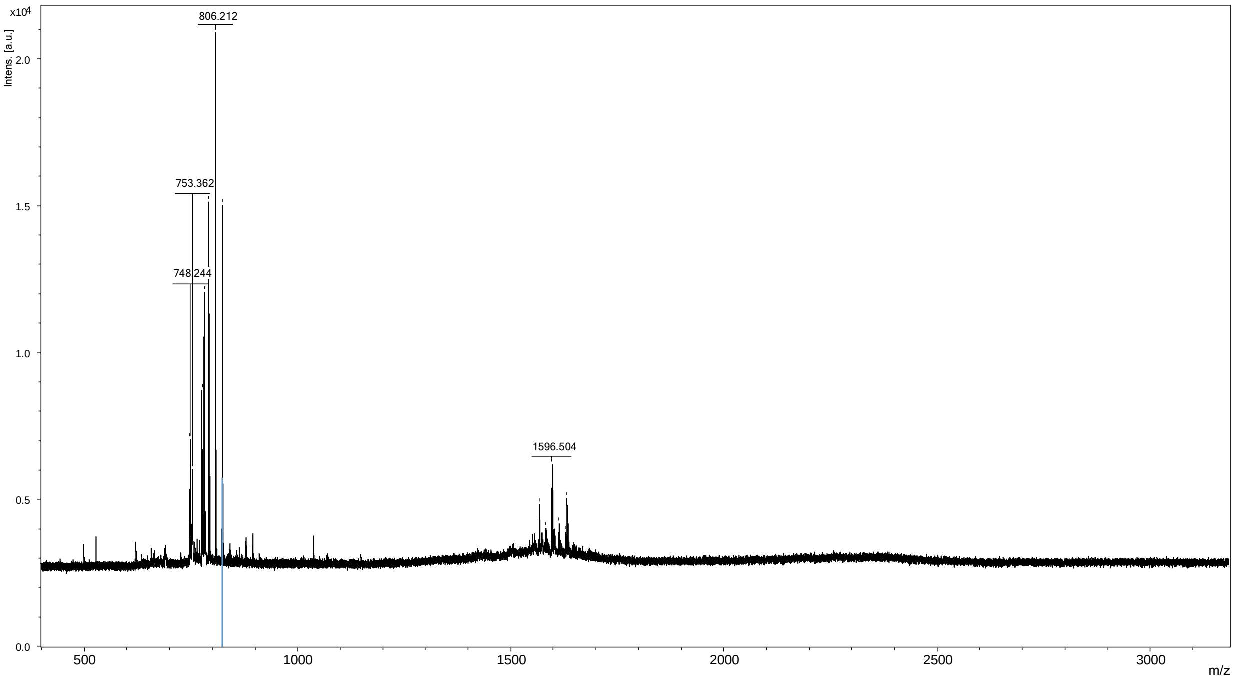

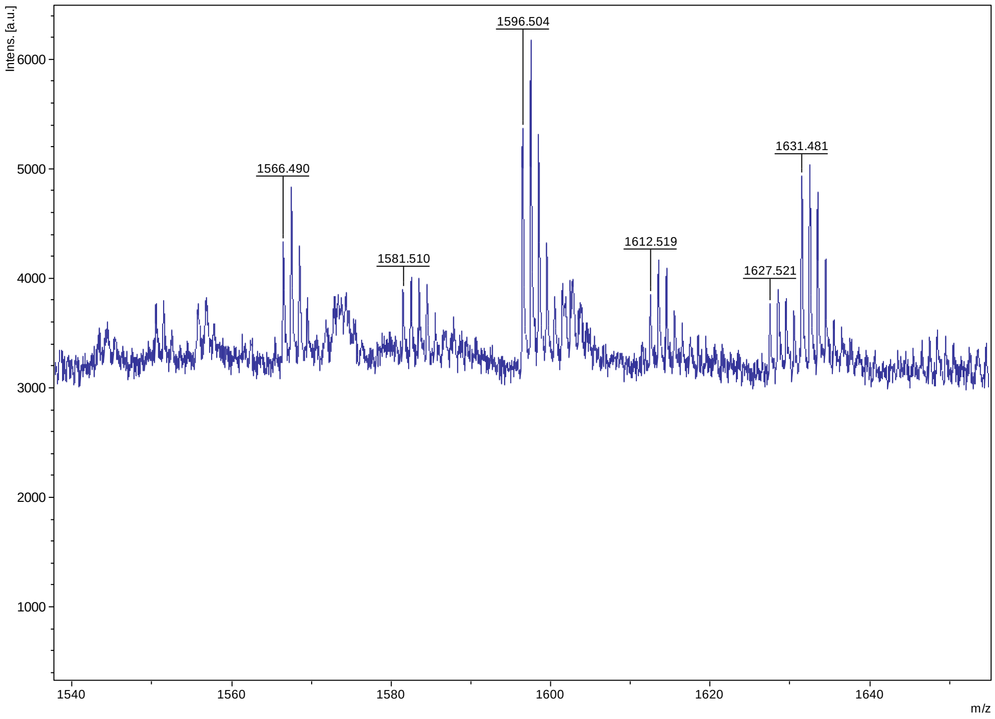

Figure S18. MALDI-TOF spectra of cobalt (II) [cobalt (III) 5,15-4-*t*-butylphenyl-10-{4-[-(2-methylpyridine)-1,2,3-triazole]}-corrole]$_2$.

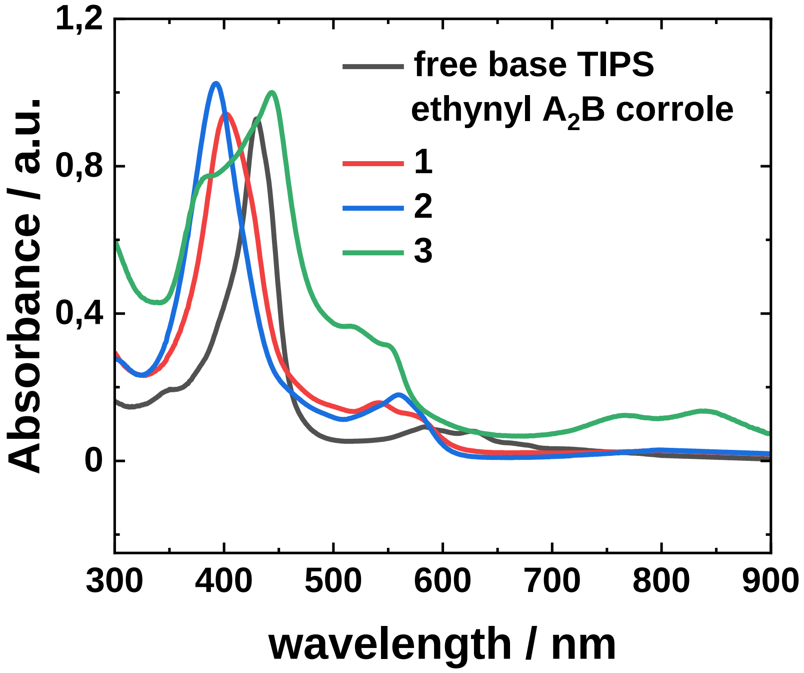

Figure S19. UV-vis spectrum of free base corrole and cobalt complexes **1, 2,** and **3** in CH_2Cl_2**.**

Table S1. Elemental identification and quantification from XPS analysis of Co^{II} TP $[Co^{III}C]_2$ **3**.

Name	Peak BE	FWHM eV	Area(P) CPS.eV	Atomic %
C1s	281.99	2.38	34046.32	84.93
O1s	529.37	1.59	6861.11	6.69
N1s	396.25	3.08	4794.41	7.51
Co2p	777.29	3.14	4867.98	0.88

Table S2. XPS analysis of Nitrogen (N1s) from Co^{II} TP $[Co^{III}C]_2$ **3**.

Name	Peak BE	Height CPS	Area CPS.eV	Area Ratio
N1s (=N-)	398.98	39.91	58.82	1
N1s (N=N)	400.98	9.91	14.6	0.25
N1s (N-C)	402.81	11.28	16.62	0.28
N1s (N-Co)	400.33	14.87	21.92	0.37

Table S3. XPS analysis of Co2p3/2 from Co^{II} TP $[Co^{III}C]_2$ **3**.

Name	Peak BE	Height CPS	Area CPS.eV	Area Ratio
Co2p (Co^{3+})	780.37	23.35	42.61	1
Co2p (Co^{2+})	782.28	12.18	22.24	0.52
Co2p (sat.-Co^{2+})	786.16	3.21	11.71	0.27

4. Electrochemical Characterization

For electrode preparation, to 1 mL ethanol (EtOH) solution containing 1.0 mg carbon black (CB) and 1.0 mg $CoTP[CoC]_2$ were added together with 10 μL Nafion. The mixture was sonicated in an ultrasonic cleaner for 1 hour. The suspension was applied to the surface of glassy carbon on RRDE (15 μL).

4.1. Procedure of Catalyst-Loaded Electrodes

Electrocatalytic ORR studies were carried out by using Bipotentiostat Pine WaveDriver 20 AFCP2 in a conventional four-electrode configuration, using a rotating-ring disk electrode (RRDE) loaded with the catalyst on the glassy carbon as the working electrode (WE 1), covered with platinum ring as working electrode 2 (WE 2), platinum wire as the counter electrode, and Hg/HgO as the reference electrode. The geometric dimensions of RRDE: platinum ring electrode (0.235 cm^2) and disk electrode (0.237 cm^2). CVs were recorded in N_2 and O_2-purged 0.1 M KOH solutions at 10 mV s^{-1} scan rate. LSVs were measured with a scan rate of 10 mV s^{-1} between 400 to 2400 rpm. The number (n) of electrons transferred during ORR was determined by K-L plots and cross-checked by RRDE[6].

From RRDE analysis, the number of electrons (n) was calculated by the formula presented below:

$$n = 4\times \frac{I_d}{I_d+\frac{I_d}{N}}$$

Also from the RRDE measurements, the H_2O_2 selectivity was obtained from the ORR polarization curves in 0.1 M KOH electrolyte (pH 13) purged with O_2 and a Pt ring was fixed at 1.2 V versus RHE. The according equation to calculate the H_2O_2 selectivity is given below:[6]

$$H_2O_2 \text{ Selectivity } (\%) = \frac{200}{1+\frac{N*I_d}{I_r}}$$

4.2. Koutechy-Levich Kinetics and Determination of n Values

Koutecky-Levich Equation: $j^{-1} = j_k^{-1} + j_d^{-1}$

j : current density, A·cm^{-2}

j_k : kinetic current density, A·cm^{-2}

$j_d = 0.62nFD_0^{2/3}v^{-1/6}C_0\omega^{1/2}$, diffusion-limited current density, A·cm^{-2}

F: Faraday's Constant, 96485 C·mol^{-1}

D_o: diffusion coefficient of O_2 in 0.1 M KOH (1.93×10^{-5} cm^2·s^{-1})

v: kinematic viscosity of the electrolyte, 0.1 M KOH, (1.09×10^{-2} cm^2·s^{-1})

C_o: saturation concentration of O_2 in 0.1 M KOH at 1 atm O_2 pressure, (1.26×10^{-6} mol·$cm^{-3)}$

ω: rotation rate, rad·s^{-1}.[7]

The electrochemical impedance spectroscopy was measured by potentiostat model Vertex.5A Ivium Technologies B.V. OER measurements were also carried out by the same compactostat. For electrocatalytic OER characterization, three-electrode configuration was used in 1 M KOH solutions by using carbon paper coated with catalyst as the working electrode, platinum wire as counter electrode and Hg/HgO as reference electrode. LSVs were measured with a scan rate of 10 mV s^{-1}. Tafel plots were calculated based on LSV data measured with 10 mV s^{-1} scan rate. The overpotential (η) was calculated:

E(RHE) = E(Hg/HgO(1M KOH)) + 0.098 + 0.059 * pH - 1.23V

For electrochemically active surface area (ECSA),

$i_c = vC_{dl}$

$$A_{ECSA} = \frac{C_{dl}}{C_s}$$

catalysts were loaded on GC electrodes without Nafion. The ECSA values can be calculated using the equations below: in which i is the charging current, v is the scan rate, and C is the specific capacitance (27 μF cm^{-2}).[8]

The equation for Faradaic efficiency:

FE% = (amount of product × n × F)/Q × 100

n = Number of electrons involved in formation of 1 product molecule
F = Faradaic constant
Q = Amount of charge passed through the working electrode

4.3. Characterization by Electrochemical Impedance Spectroscopy (EIS) - Method

The electrochemical impedance spectroscopy (EIS) was measured using a potentiostat model Vertex.5A Ivium Technologies B.V. The impedance spectrum was recorded for all experiments in the frequency range of 10^6 Hz to 0.1 Hz with a perturbation amplitude of 10 mV. The aim of this characterization is the investigation of Co^{II} TP $[Co^{III}C]_2$ **3** for the ORR and OER electrolysis. To realize the measurements, 0.1 M KOH and 1M KOH were employed for ORR and OER, respectively. For the determination of the cell parameters in the case of ORR, first two platinum electrodes were measured in a one cell compartment with the corresponding electrolyte as a control experiment. Next, one platinum electrode was replaced by a glassy carbon electrode as a working electrode. Finally, the glassy carbon loaded with the electrocatalyst was set as working electrode, to characterize the complete electrochemical cell by impedance spectroscopy. All fitted and calculated impedance data are summarized in the table S4.

For the EIS measurements of OER, first two platinum electrodes were measured in a one cell compartment with the corresponding electrolyte as a control experiment to determine the electrolyte resistance. Afterwards, the setup was transferred to a H-cell configuration with Nafion membrane inbetween. By this way the resistance of the membrane is found and immediately substracted from the electrolyte resistance. Next, one platinum electrode was replaced by a carbon paper electrode as a working electrode. Finally, the carbon paper coated with the Co^{II} TP $[Co^{III}C]_2$ **3** was set as working electrode, to evalute the complete electrochemical cell by EIS. All fitted and calculated impedance data are summarized in the table S5. The bode plot for the two-electrode system is shown in the manuscript. All resistance values for each cell components, i.e., electrolyte solution, membrane, carrier electrode are shown in tables S4 and S5 for ORR and OER cell systems, respectively. Based on EIS, the applied electrochemical cells were characterized in detail indicating negligible losses of the systems.

Table S4. Cell parameter extracted via electrochemica impedance measurements for ORR.

WE	CE	R_{Sol} / Ω	$R_{carrier}$ / Ω	R / Ω	R_{Me} / Ω	$C_{Co\ [CCPT]2}$ / F	CPE-T	CPE-P
Pt	Pt	2.92E+00	3.840E+05	-	2.23E01	-	1.107E-04	9.203E-01
Cp	Pt	2.92E+00	6.91E+04	-	2.23E01	-	3.214E-05	9.163E-01
Co^{II} TP $[Co^{III}C]_2$ 3	Pt	2.92E+00	7.193E+04	2.678E+01	2.23E01	2.045E-06	6.613E-05	9.531E-01

Table S5. Cell parameter extracted via electrochemical impedance measurements for OER.

WE	CE	R_{Sol} / Ω	$R_{carrier}$ / Ω	R / Co $[CCPT]_2$ Ω	R_{Me} / Ω	$C_{Co\ [CCPT]2}$/ F	CPE-T	CPE-P
Pt	Pt	4.11E+01	9.85E+04	-	4.05E+02	-	1.241E-04	8.562E-01
GC	Pt	4.11E+01	5.20E+04	-	4.05E+02	-	2.930E-05	1.025E+00
Co^{II} TP $[Co^{III}C]_2$ 3	Pt	4.11E+01	9.82E+04	9.13E+02	4.05E+02	2.850E-05	5.430E-05	8.649E-01

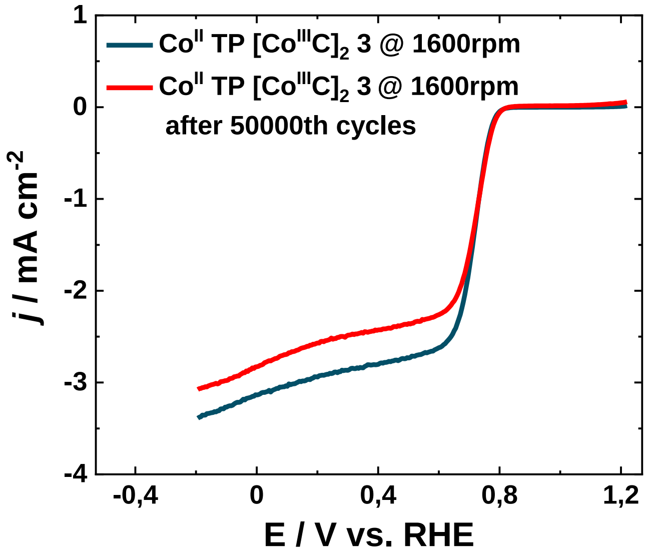

Figure S20. Linear-sweep voltammograms (LSVs) of Co^{II}TP[Co^{III}C]$_2$ **3** before and after 50000 cycles of stability test in 0.1 M KOH.

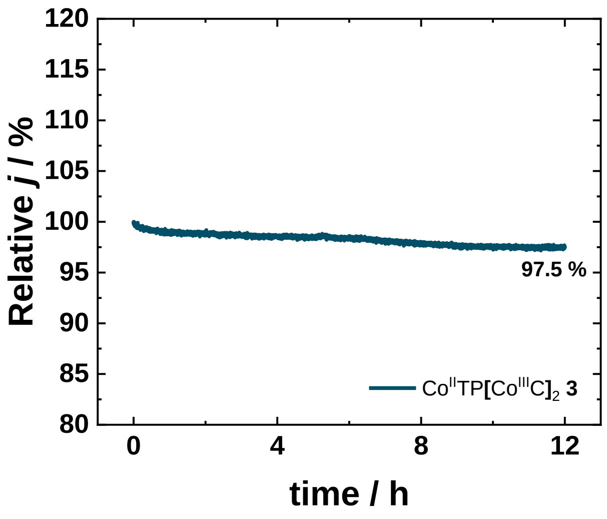

Figure S21. Chronoamperometric measurements of Co^{II}TP[Co^{III}C]$_2$ **3** under ambient atmosphere at 1600 rpm to prove the long-term stability of the catalyst.

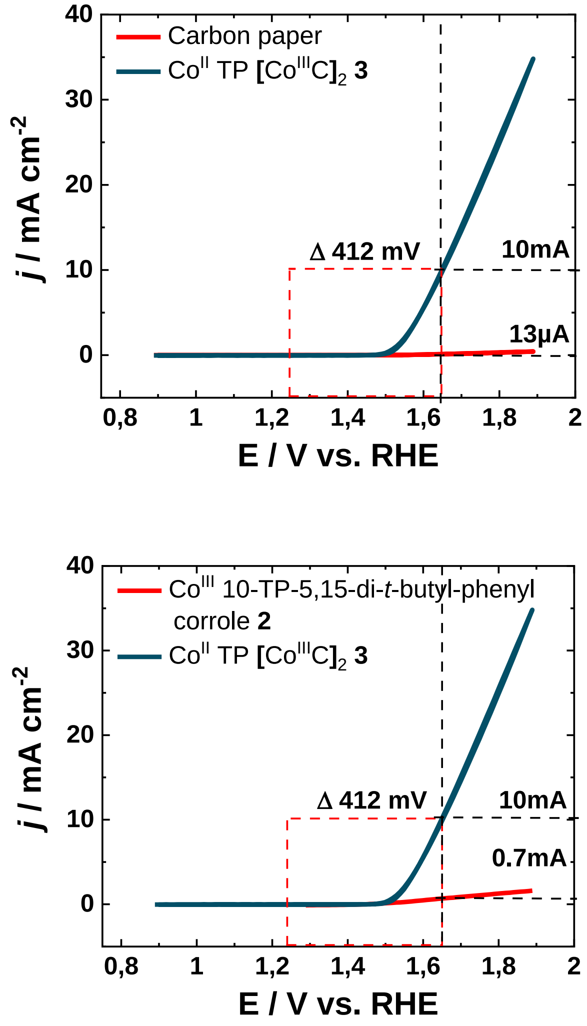

Figure S22. Linear-sweep voltammograms (LSVs) of Co^{II} Pyridine triazole and Co^{II}TP [Co^{III}C]$_2$ **3** in 1 M KOH.

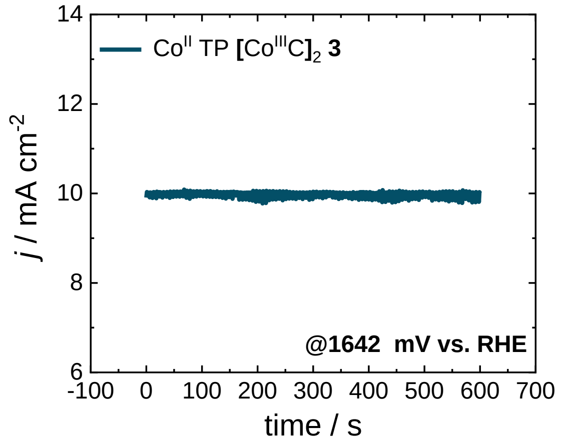

Figure S23. Chronoamperometric measurements Co^{II}TP[Co^{III}C]$_2$ **3** at 1642 mV to calculate the faradaic efficiency.

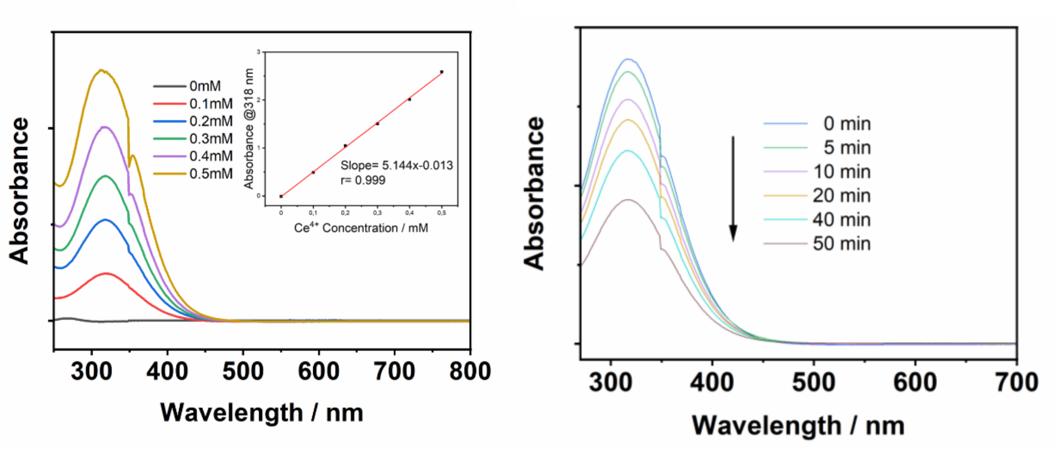

Figure S24. Ceric sulfate titration of H_2O_2.

The ceric sulfate titration of H_2O_2 reaction: 2 Ce^{4+} + H_2O_2 → 2 Ce^{3} + 2 H^+ + O_2.

Ce^{4+} has a peak absorbance at 319 nm while Ce^{3+} is colorless.
We prepared a series of standard Ce^{4+} solutions (up to 0.5 mM). Absorption spectroscopy of standard Ce^{4+} solutions was performed at 319 nm, and a calibration curve was generated.
Abs = ε × l × [Ce^{4+}] where Abs is the absorbance at 319 nm, [Ce^{4+}] is the Ce^{4+} concentration (mM), ε is the molar absorptivity of Ce^{4+} (mM-1 cm-1), and l is the path length (1 cm)

$[Ce^{4+}]_{before}$ = Abs_{before} /ε × l
$[Ce^{4+}]_{after}$ = Abs_{after} /ε × l

H_2O_2 concentration (mM) in the 50-µL aliquot = (4 × $[Ce^{4+}]_{before}$ - 4.05 × $[Ce^{4+}]_{after}$) /(2 × 0.05)
where Abs_{before} and Ab_{safter} are the absorbances of Ce4+ stock solution at 319 nm before and after injecting H_2O_2-containing aliquot.
Cumulative Faradaic efficiency (%) = (100 × 2 × 96485 × Cumulative H_2O_2 yield (mol) / ∫(I dt)
Cumulative H_2O_2 selectivity (%) = 200 / {1 +[∫ I dt/(2 × 96485 × Cumulative H_2O_2 yield (mol))]}
Cumulative H_2O_2 selectivity (%) = 200/ 1 + (100/ Cumulative Faradaic efficiency (%))

A. <u>60 minutes calculations</u>

n (mol) product: 15.3171 µmol
n (mol) catalyst: 6.517 nm
Cumulative Faradaic efficiency= 50%
Cumulative H_2O_2 selectivity= 66%
TON= 2350 considering each molecule a single catalyst site or 1175 considering each site as 2 catalyst sites for each Co^{3+}
TOF=0.652 S^{-1} or 0.326 s^{-1}

B. <u>5 minutes calculations</u>

n (mol) product: 2.5272 µmol
n (mol) catalyst: 6.517 nm
Cumulative Faradaic efficiency= 81.2%
Cumulative H_2O_2 selectivity=90%
TON= 387.7 or 193.85
TOF= 1.2926 S^{-1} or 0.6481 s^{-1}

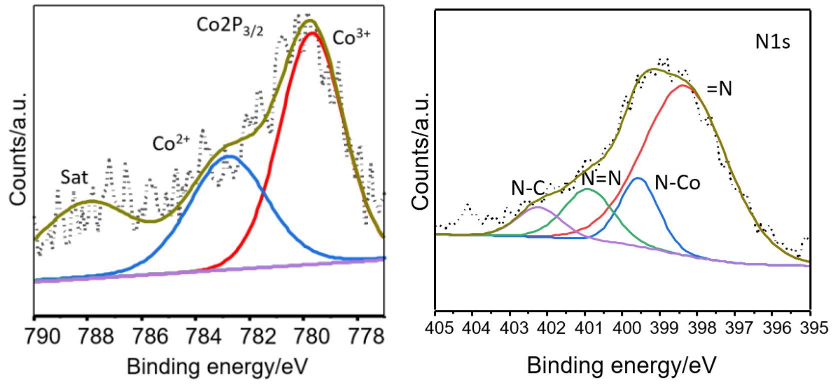

Figure S25. XPS narrow scans for Cobalt ($Co2p_{3/2}$) and Nitrogen (N1s) present in $Co^{II}TP[Co^{III}C]_2$ **3** on carbon paper after electrocatalytic oxygen evolution measurements.

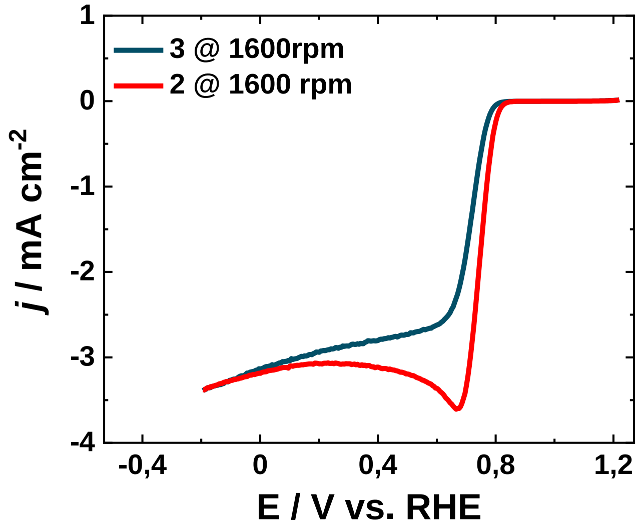

Figure S26. Electrochemical ORR catalytic performance of complex **2**. Linear sweep voltammograms (LSVs) of **2** and Pt ring at 1600 rpm sweep rate: 10 mV/s measured on RRDE.

5. References

[1] L. M. Urner, M. Sekita, N. Trapp, W. B. Schweizer, M. Wörle, J.-P. Gisselbrecht, C. Boudon, D. M. Guldi, F. Diederich, *European Journal of Organic Chemistry* **2015**, *2015*, 91-108.

[2] aG. A. Brito, F. Della-Felice, G. Luo, A. S. Burns, R. A. Pilli, S. D. Rychnovsky, M. J. Krische, *Org Lett* **2018**, *20*, 4144-4147; bK. Ueta, K. Naoda, S. Ooi, T. Tanaka, A. Osuka, *Angew Chem Int Ed Engl* **2017**, *56*, 7223-7226.

[3] S. K. Mamidyala, M. A. Cooper, *Chem Commun (Camb)* **2013**, *49*, 8407-8409.

[4] B. Koszarna, D. T. Gryko, *The Journal of Organic Chemistry* **2006**, *71*, 3707-3717.

[5] D. Urankar, B. Pinter, A. Pevec, F. De Proft, I. Turel, J. Košmrlj, *Inorganic Chemistry* **2010**, *49*, 4820-4829.

[6] J. Song, J. M. Yu, J. H. Ahn, H. Cho, J. Oh, Y. S. Kim, J. Kim, M. Ko, S. h. Lee, T. J. Shin, H. Y. Jeong, C. Yang, J. H. Lee, J. W. Jang, S. Cho, *Advanced Functional Materials* **2022**, *32*.

[7] aB. B. Blizanac, P. N. Ross, N. M. Marković, *The Journal of Physical Chemistry B* **2006**, *110*, 4735-4741; bP. Singh, D. A. Buttry, *The Journal of Physical Chemistry C* **2012**, *116*, 10656-10663.

[8] C. C. L. McCrory, S. Jung, J. C. Peters, T. F. Jaramillo, *Journal of the American Chemical Society* **2013**, *135*, 16977-16987.
